# Recovery of mouse growth hormone from *E. coli* inclusion bodies using a mild solubilisation and repeated freeze–thaw approach

**DOI:** 10.1007/s11033-025-10685-y

**Published:** 2025-06-23

**Authors:** Minah Kim, Ries J. Langley, Jo K. Perry, Yue Wang

**Affiliations:** 1 Liggins Institute, University of Auckland, 85 Park Rd, Private Bag 92019, Auckland, 1142 New Zealand; 2 Department of Molecular Medicine and Pathology, University of Auckland, Auckland, 1023 New Zealand; 3 Maurice Wilkins Centre for Molecular Biodiscovery, Auckland, 1023 New Zealand

**Keywords:** Recombinant protein expression, Mouse growth hormone, Inclusion body, Mild solubilisation

## Abstract

**Background:**

Recombinant mouse GH (mGH) is a critical tool for investigating GH-GH receptor (GHR) interactions in rodent models. However, while numerous methods exist for producing human GH, detailed protocols for the expression and purification of recombinant mGH remain scarce in the literature.

**Methods:**

We developed a method for refolding mGH from inclusion bodies produced in *Escherichia coli* (*E. coli*). Recombinant mGH was fused with a N-terminal thioredoxin (Trx) tag and was expressed as inclusion body proteins in *E. coli* when induced at 30 °C. Inclusion bodies were isolated and solubilised under mild conditions (50 mM Tris–HCl, 2 M urea, pH 10.5) and freeze-thawed.

**Results:**

Three rounds of freeze-thawing improved solubilisation when compared to a single round. Subsequently, recombinant mGH underwent Trx-tag removal and was purified using anion exchange chromatography to achieve a purity of 98%. Purified mGH displayed circular dichroism spectral profiles comparable to that of commercially sourced mGH, confirming the preservation of the secondary structure in refolded mGH. Bioactivity was confirmed using a Ba/F3-mGhr cell viability assay and showed that refolded mGH had comparable bioactivity to commercially sourced mGH. Bioactivity was also assessed by measuring activation of mGH receptor signal transduction in B16-F10 mouse melanoma cells by phosphorylated STAT5 western blot analysis.

**Conclusions:**

We present an efficient and cost-effective protocol for mGH production. This repeated freeze–thaw approach may have broad application for other proteins expressed in the form of inclusion bodies for which low yields are observed following a single round of freeze–thaw.

**Supplementary Information:**

The online version contains supplementary material available at 10.1007/s11033-025-10685-y.

## Introduction

Growth hormone (GH) is a 22 kDa pituitary-secreted hormone critical for a multitude of physiological processes, including promotion of longitudinal growth, regulating metabolism, and maintaining muscle mass [1]. In addition to actions in normal physiology, its involvement in the pathogenesis of various diseases and conditions, such as cancer, diabetes, and aging, has become increasingly evident [2–4].

Recombinant GH can be produced in *Escherichia coli* (*E. coli)* and eukaryotic expression systems. Production and purification methods for GH from numerous species (including human, bovine, and fish) have been widely reported [4]. However, to date, there is a paucity of published literature detailing large-scale production and purification of recombinant mouse GH (mGH). mGH has been produced utilising fed-batch fermentation methods. However, when expressed in *E. coli*, soluble expression is poor, with most protein in the form of inclusion bodies [5, 6]. Although providing the benefit of enriching for the recombinant protein-of-interest, often at high concentration and purity, inclusion bodies can also hinder efficient recombinant protein production by necessitating additional steps to denature and refold the proteins. Furthermore, the optimal conditions for inclusion body recovery, such as denaturant concentrations and the refolding conditions, vary by protein. A freeze-thawing method, was recently reported to improve solubilisation of several proteins from inclusion bodies [7].

Although available commercially, the high cost of recombinant mGH impedes accessibility when larger quantities are required. Here we optimised a cost-effective method to produce active recombinant mGH in *E. coli*. Different expression and purification methods were explored, to solubilise the protein from inclusion bodies via freeze-thawing, protein refolding, and anion exchange chromatography. In addition, we report an effective modification to the freeze-thawing method which enhances the yield of soluble mGH protein from inclusion bodies.

## Methods

### Cell lines and materials

The Ba/F3-mGhr cell line (established in-house) stably overexpresses mGH receptor. Cells were cultured in RPMI medium supplemented with 5% fetal bovine serum (FBS; Moregate BioTech, New Zealand), 100 U/mL penicillin, 100 µg/mL streptomycin, 1% glutaMAX, and 25 ng/mL mGH, and incubated in a humidified incubator at 37 °C, 5% CO_2_.

The B16-F10 mouse melanoma cell line was provided by the Auckland Cancer Society Research Centre cell line bank. Cells were cultured in MEM-α medium supplemented with 5% FBS, 100 U/mL penicillin, 100 µg/mL streptomycin, and 0.25 µg/mL amphotericin B and were maintained in a humidified incubator at 37 °C, 5% CO_2_.

Commercial recombinant mGH was obtained from NHPP Harbor-UCLA Research and Education Institute.

### Construction, expression, and purification of recombinant mGH

#### Construction of the expression plasmid

The *mGh* DNA sequence (NCBI reference seq: NM_008117.3) was codon optimised for *E. coli* expression system and synthesised by TWIST Bioscience. The DNA fragment was amplified by polymerase chain reaction (PCR) and amplified DNA fragments were ligated into the pET32a.3C vector which contains an N-terminal thioredoxin (Trx) tag and a human rhinovirus 3C protease cleavage site between the Trx-tag and mGH (Fig. S1). The pET32a.3C-mGh plasmid was verified by Sanger sequencing.

#### Expression and purification of recombinant mGH

The pET32a.3C-mGh plasmid was transformed into AD494(DE3)*pLysS E. coli* via heat shock and colonies were grown on LB agar. A single colony was cultured in 1 L LB media (containing 100 µg/mL ampicillin, 34 µg/mL chloramphenicol, and 15 µg/mL kanamycin) in a shake flask at 225 rpm. At optical density 600 nm (OD_600_) of 0.4, recombinant Trx-mGH expression was induced using 0.1 mM isopropyl-β-D-thiogalactopyranoside (IPTG) at 225 rpm for 4 h at 37 °C and 30 °C, and 18 h at 18 °C. Bacterial cells were harvested by centrifugation at 6,000 *g* for 20 min. The cell pellets were resuspended in cold lysis buffer (1 X PBS, pH 7.4, with 150 mM NaCl, 1% Triton X-100, 10% glycerol, 1 mM PMSF) and stored at − 80 °C.

For protein extraction, cell lysates were sonicated using 200 W (60 × 1 s pulses) using a Vibra-Cell™ sonicator (Sonics & Materials, Newtown, CT, USA), followed by centrifugation at 18,500 g for 20 min. The supernatant (soluble proteins) and the pellet (inclusion bodies) fractions were analysed by sodium dodecyl sulphate polyacrylamide gel electrophoresis (SDS-PAGE) and Coomassie Blue staining to visualise recombinant protein expression.

#### Inclusion body extraction, solubilisation, and refolding

The inclusion body pellet was washed extensively by repeating the sonication-PBS wash-centrifugation steps three times. The inclusion body pellets were then resuspended in inclusion body solubilisation buffers at different pH values (50 mM Tris–HCl, pH 8.5, 9.5, and 10.5) and containing different concentrations of urea (0–8 M), and were frozen at -20 °C. The solubilised inclusion body solutions were thawed and centrifuged at 18,500 × *g* for 20 min at 4 °C. Some solubilised inclusion body solutions underwent two additional cycles of freeze-thawing. Supernatants were analysed by SDS-PAGE and dialysed in 50 mM Tris–HCl, pH 8.5, with redox reagents (1 mM cystamine and 5 mM cysteamine) and 10% glycerol for 48 h at 4 °C to refold the proteins.

#### Protein purification

Dialysed fractions containing Trx-mGH were incubated with 3C protease at 4 °C overnight to remove the Trx tag from mGH. mGH was then purified by anion-exchange chromatography using the Mono Q 5/50 column (Cytiva). Samples equilibrated in 50 mM Trix-HCl, pH 8.5 were eluted over a 10-column volume linear gradient of 0.3 M NaCl. Fractions forming the peak containing the recombinant mGH were pooled and dialysed in PBS buffer. Protein concentrations were determined using a Pierce bicinchoninic acid (BCA) assay with bovine serum albumin (BSA) as a standard (ThermoFisher). Protein purity was assessed by SDS-PAGE followed by Coomassie Blue staining. Densitometric analysis was performed using the Image Lab 6.1 (Bio-Rad), which quantifies band intensities based on pixel density. The purity of the target protein was calculated as the percentage of the target band intensity relative to the total lane intensity, providing an estimate of protein purity.

### Circular dichroism (CD) spectroscopy

Far-UV CD spectra were recorded using a Chirascan CD Spectrometer in the wavelength range of 200 – 280 nm at 20 °C. Recombinant mGH was analysed in a 1 mm path length cuvette at the molarity of 2.5 µM in PBS. Three independent spectra were acquired for each sample and the averages were plotted.

### Cell viability assay

Ba/F3-mGhr cells were serum starved overnight and seeded at 30,000 cells/well in 80 µL in a 96-well plate. Cells were treated with 20 µL of serial dilutions of the recombinant mGH (0–400 ng/mL) in serum-free media for 48 h. Resazurin dye (5 µL of 5 mg/mL stock) was added and further incubated for 2 h. Fluorescence was measured at 530–560 nm excitation and 590 nm emission wavelengths.

### Western blot

B16-F10 cells were serum-starved for 6 h and incubated with different concentrations of mGH for 30 min, followed by an ice-cold PBS wash. Cell lysates were collected using cold lysis buffer (1% Triton X-100, 150 mM NaCl, 10% glycerol, 50 mM Tris–HCl pH 7.4, 2 mM EDTA, 100 mM NaF, 1 mM Na_3_VO_4_, HALT protease inhibitor, 1 mM PMSF), and centrifuged at 18,500 g for 20 min. The supernatant was collected and electrophoresed on a 7.5% SDS-PAGE gel, then transferred to a polyvinylidene fluoride (PVDF) membrane. Membranes were blocked with 5% milk in TBS-T solution, then incubated with primary antibody (phospho-STAT5 alpha (TYR694), Invitrogen, 716,900; total STAT5 (Santa Cruz Biotechnology, sc-835) and β-actin (Sigma Chemical Company, A1978). The immunoreactive bands were detected by enhanced chemiluminescence.

### Enzyme-linked immunosorbent assay

Microtiter plates were coated with antigens (mGH and Trx-mGH) diluted in phosphate buffer at 2 µg/mL and 4 µg/mL, respectively, and incubated overnight at 4 °C. The coated plates were washed and blocked with 1% BSA, followed by 1 h incubation with 100 µL primary antibody (anti-mGH mAb or control IgG) at 2 µg/mL and 0.5 h incubation with conjugated secondary antibody. Samples were incubated with TMB for 30 min, protected from light, and absorbance was read at 450 nm and 590 nm.

### Statistical analysis

GraphPad Prism (version 10.1.2) software was used for statistical analyses. A p-value of < 0.05 was accepted as statistically significant. The half-maximal effective concentration (EC_50_) was obtained from fitting a sigmoidal dose–response model “Log(agonist) vs response – Variable slope (four parameters)”. Unless otherwise stated, experiments were repeated at least three times with a representative figure shown.

## Results

### Optimisation of recombinant mGH expression

The codon-optimised *mGh* gene was cloned into the pET32a.3C vector which contains an N-terminal Trx-His6 tag and a human rhinovirus 3C protease cleavage site (Figure S1). The Trx fusion tag was selected as it has been shown to improve solubility of proteins in *E. coli* strain, AD494, and enhances the formation of disulfide bonds in the bacteria [8, 9]. The pET32a.3C-mGh plasmid was transformed into AD494(DE3)*pLysS E. coli* and protein expression was induced with 0.1 mM IPTG at 37 °C, 30 °C, or 18 °C.

**Fig. 1:**
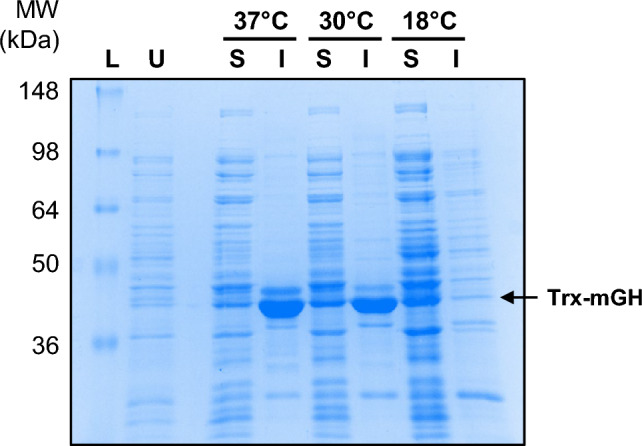
Expression of recombinant Trx-mGH in *E. coli*. *E. coli* harbouring pET32a.3C-mGh were induced with IPTG at either 37 °C, 30 °C for 4 h, or 18 °C for 18 h, and the soluble and insoluble protein from each condition were examined for recombinant Trx-mGH expression (~ 40 kDa, indicated by arrow). U: uninduced bacterial lysates, S: soluble protein, P: pellets, MW: molecular weight. Equal volumes of samples were loaded on a 12.5% SDS-PAGE gel and stained using Coomassie blue

Solubility of the Trx-mGH protein was dependent on the induction temperature. Protein induction at 37 °C and 30 °C caused the majority of recombinant Trx-mGH to accumulate as insoluble protein aggregates in inclusion bodies. Lowering the induction temperature to 18 °C increased the yield of soluble protein (Fig. 1). Soluble protein expressed at 18 °C was subjected to 3C cleavage and purified using Ni–NTA affinity chromatography (Figure S2A). The purified Trx-mGH, along with commercially sourced mGH, were both recognised by a mGH-specific antibody (anti-mGH mAb) (Figure S2B). The final yield of purified recombinant mGH obtained by induction at 18 °C was 0.3 mg per litre of bacterial culture.

Autoinduction of protein expression was also attempted to improve the yield of soluble Trx-mGH. Transformed *E. coli* were cultured in AIM-2YT broth autoinduction medium (Formedium Ltd.) at 18 °C for 6 days and the culture samples were collected every 24 h. However, there was no major improvement in soluble protein expression, as demonstrated by SDS-PAGE analysis (Figure S3A & B). Expression of recombinant mGH was confirmed by western blot but the amount of mGH decreased on each subsequent day of measurement (Figure S3C).

We also attempted to express mGH without a Trx-tag in *E. coli* as it would simplify the purification process by eliminating the need for tag cleavage*.* However, this approach was less successful with very low protein expression observed (data not shown), suggesting that fusion to a protein such as Trx may be necessary to improve mGH expression in this setting.

### Refolding mGH protein from inclusion bodies

**Fig. 2:**
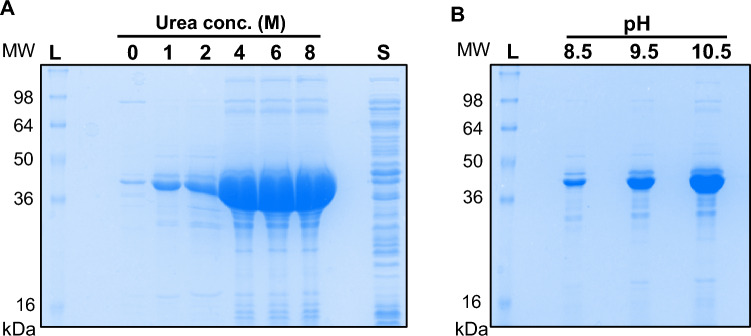
Optimisation of the inclusion body solubilisation method for improved yield of Trx-mGH protein. **A** SDS-PAGE analysis of Trx-mGH inclusion bodies solubilised in 0–8 M urea in 50 mM Tris–HCl pH 8.5 by the freeze–thaw method. S: supernatant from cytoplasmic proteins. **B** The effect of pH of the 2 M urea buffer on the solubility of Trx-mGH inclusion bodies. Equal volumes of samples were loaded on each 12.5% SDS-PAGE gel and stained using Coomassie blue. L: Ladder

The majority of Trx-mGH protein aggregated as insoluble inclusion bodies when induced at 30 °C. Therefore, solubilisation using a denaturant such as urea was required. Pellets of inclusion bodies were washed extensively in PBS and then resuspended in equal volumes of 50 mM Tris–HCl pH 8.5 with different concentrations of urea, ranging from 0–8 M. Following a single freeze–thaw cycle, supernatants containing solubilised protein were analysed by SDS-PAGE. Efficient solubilisation of protein in the inclusion bodies was observed with buffers containing 4, 6, and 8 M urea (Fig. 2A).

Next, we investigated whether altering the pH might improve inclusion body resolution at lower urea concentrations as milder concentrations of denaturants ensure preservation of the proteins’ secondary structure during the solubilisation process [10]. Inclusion bodies were resuspended in 50 mM Tris–HCl, 2 M urea buffer at pH 8.5, 9.5, and 10.5 and assessed by SDS-PAGE. Increasing the pH value of the 2 M urea buffer from 8.5 to 10.5 significantly improved the solubilisation (Fig. 2B), indicating that solubility of Trx-mGH inclusion bodies was pH-dependent with optimal solubilisation observed at a pH value of 10.5.

**Fig. 3:**
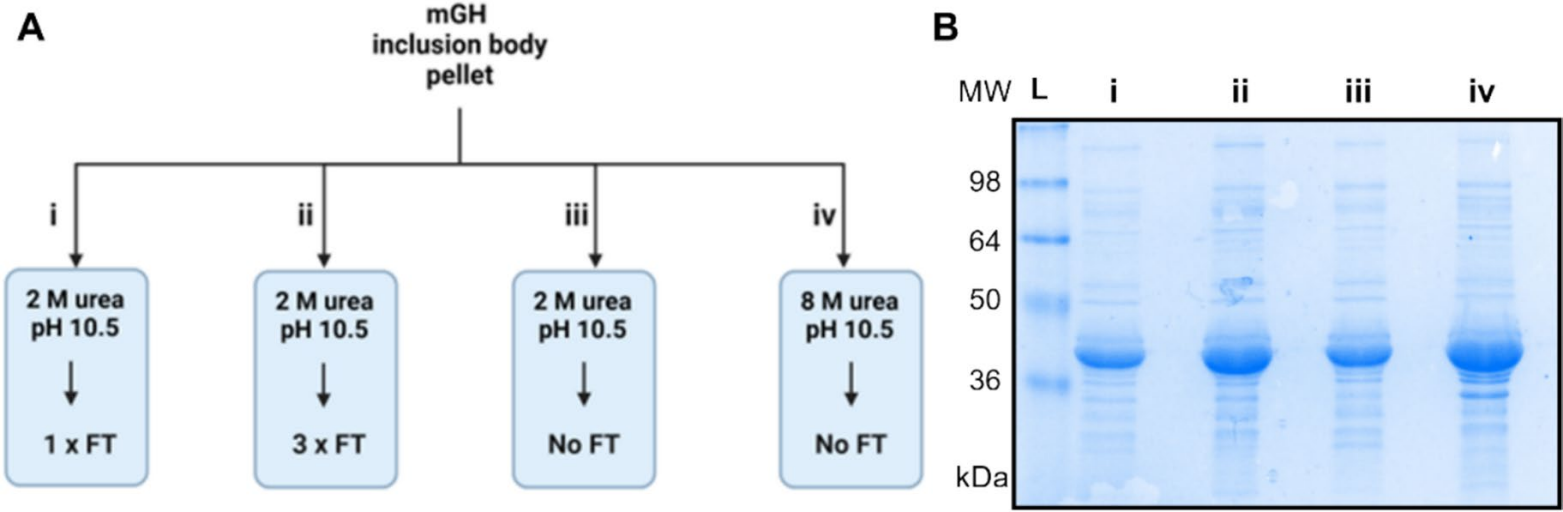
Optimising the inclusion body solubilisation method for improved yield of Trx-mGH protein. **A** Schematic diagram of inclusion body solubilisation methods. Lane i, 2 M urea with one freeze–thaw cycle; Lane ii, 2 M urea with three freeze–thaw cycles; Lane iii, 2 M urea without freeze–thaw; Lane iv, 8 M urea without freeze–thaw. **B** SDS-PAGE analysis of the solubilised inclusion bodies from methods in (A). Equal volumes of samples were loaded on 12.5% SDS-PAGE gels. L: Ladder; FT: freeze–thaw. A single repeat of this experiment was conducted

The impact of the number of freeze–thaw cycles on inclusion body solubilisation was also assessed. Trx-mGH inclusion body pellets (from equal volumes of culture medium) were resuspended in equal volumes of resuspension buffer (50 mM Tris–HCl, 2 M urea, pH 10.5) and were subjected to either one or three cycles of freeze–thaw at − 20 °C or solubilised at 4 °C overnight (Fig. 3A). We found that while a single round of freeze–thaw did not enhance solubility compared to the traditional solubilisation method, increasing the number of freeze–thaw cycles from one to three improved the solubilisation of Trx-mGH, thus demonstrating the benefit of repeated freeze–thaw cycles. Inclusion bodies solubilised in 2 M urea using three rounds of freeze–thaw achieved solubility levels close to those obtained with 8 M urea, which typically results in complete solubilisation (Fig. 3B).

**Fig. 4:**
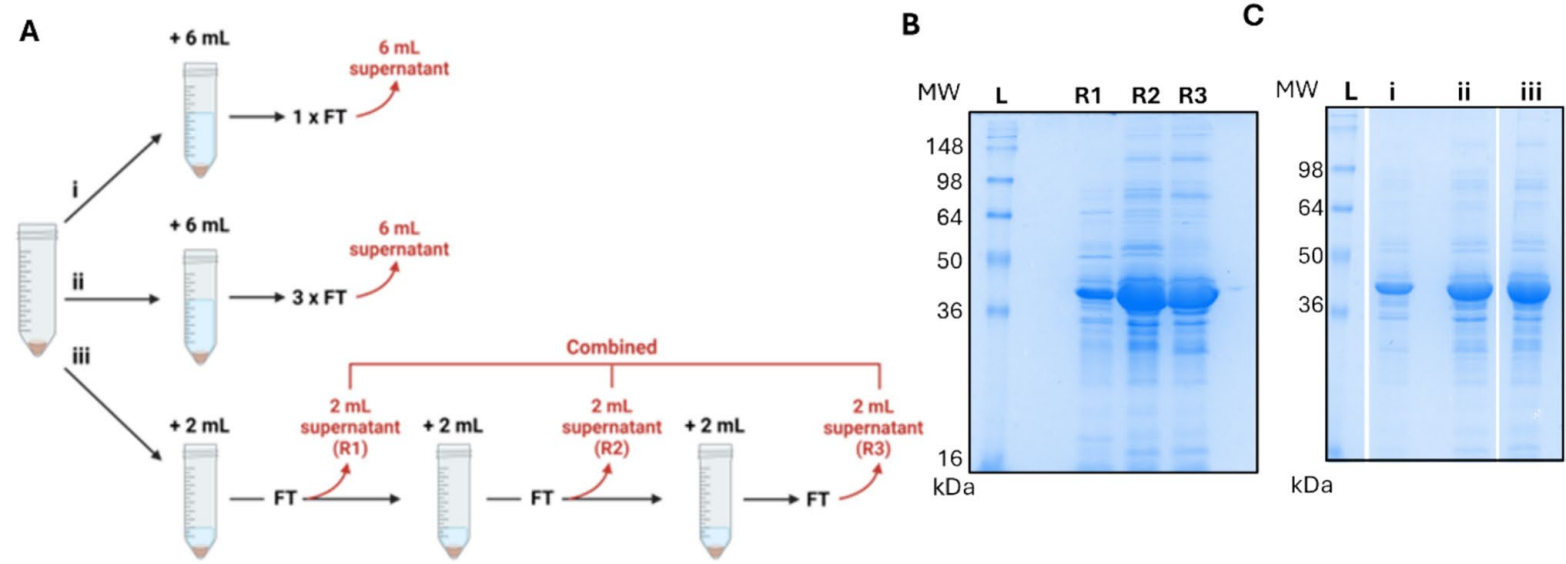
Solubilisation of inclusion body using multiple freeze–thaw cycles with buffer replacement. **A** Schematic diagram showing the different inclusion body freeze–thaw methods. **B** SDS-PAGE analysis of the solubilised inclusion bodies from the three subsequent rounds of suspension in 2 mL suspension buffer (50 mM Tris–HCl, 2 M urea, pH 10.5 buffer), freeze-thawing, and supernatant collection (R1: round 1, R2: round 2, R3: round 3). Equal volumes of samples were loaded on 12.5% SDS-PAGE gels. **C** SDS-PAGE analysis of the solubilised inclusion bodies using different strategies. Lane i, inclusion bodies resuspended in 6 mL suspension buffer and 1 freeze–thaw cycle; Lane ii, resuspended in 6 mL suspension buffer and 3 freeze–thaw cycles; Lane iii, the pool of R1, R2 and R3. L: Ladder; FT: freeze–thaw

An additional approach for solubilising Trx-mGH was also taken where the volume of resuspension buffer (6 mL) was evenly divided over the three freeze–thaw cycles (2 mL per cycle). Following each round of freeze–thaw, the supernatant was collected, and 2 mL of fresh buffer was added (Fig. 4A&B). The three supernatants (R1, R2, and R3) were analysed by SDS-PAGE (Fig. 4B). R1 had the lowest level of solubilised Trx-mGH, with increased solubilisation observed in supernatants R2 and R3. The three supernatants were pooled and this method was compared with one freeze thaw or three freeze-thaws without buffer replacement (Fig. 4A and C, i and ii). Using three cycles of freeze–thaw with 2 mL buffer replacement appeared to slightly improve protein solubilisation (Fig. 4A and C, iii), but we note variability across repeat experiments so further investigation would be required to confirm this. As three cycles of freeze–thaw without buffer replacement also increased solubilisation (Fig. 3A ii and 4A ii) and was less labour intensive, this approach was considered to be more practical.

### Purification of recombinant mGH

**Fig. 5:**
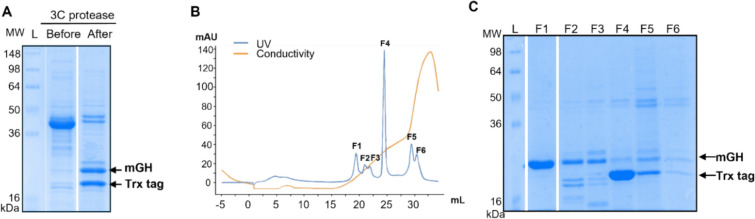
Purification of refolded recombinant mGH using anion-exchange chromatography (Mono Q). **a** Removal of the Trx tag from mGH by 3C protease cleavage and confirmed by SDS-PAGE. Molecular weights of mGH and the Trx tag are 22 kDa and 18 kDa, respectively. **b** Anion exchange chromatography elution profile. Eluted fractions 1–6 (F1-F6) were collected separately. mAU: milli-absorbance units. **c** SDS-PAGE analysis of the elution fractions F1-F6. The F1 peak contained the majority of the refolded mGH, with a purity of 98%

Trx-mGH solubilised using method iii (three cycles of freeze–thaw with 2 mL buffer replacement) was refolded by dialysing in 50 mM Tris–HCl pH 8.5 with 1 mM cystamine and 5 mM cysteamine. The Trx-tag was then removed by digestion with 3C protease (Fig. 5a). Purification of mGH from the cleaved Trx-tag and other impurities was performed by anion exchange chromatography (Mono Q) and the eluted fractions were analysed on SDS-PAGE (Fig. 5b, 5c). The majority of refolded mGH was eluted in fraction 1 (F1, the first major peak) with a purity of 98% and was also detected in smaller amounts in F2 and F3 (Fig. 5c). The final yield of recombinant mGH obtained from the F1 peak from anion exchange chromatography was 3.7 mg from 1 L of culture medium. This was tenfold higher than mGH purified from the soluble fraction after induction at 18 °C overnight.

### Secondary structure analysis of refolded mGH

**Fig. 6:**
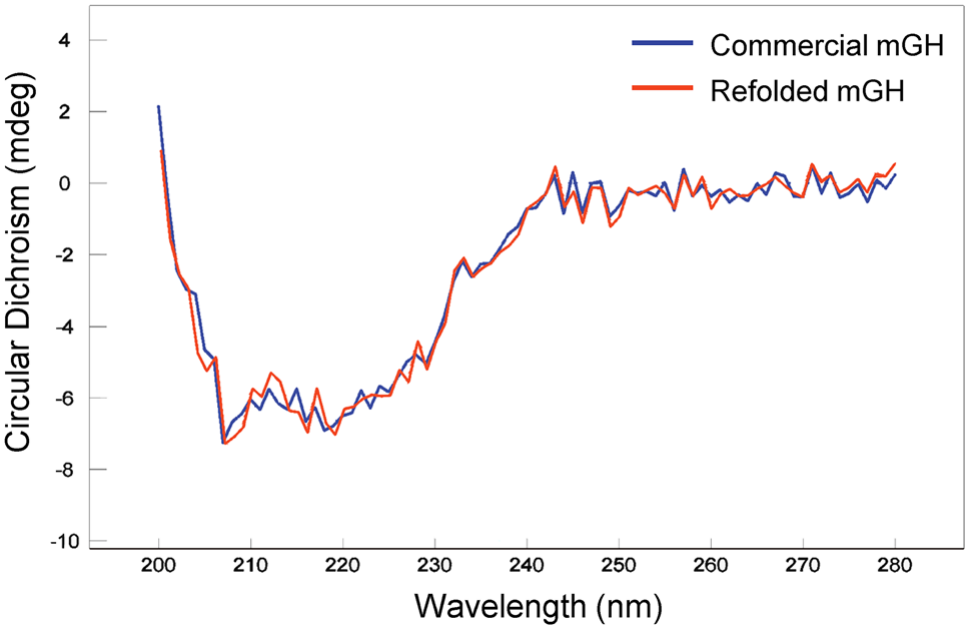
CD spectra assessment of commercial and refolded mGH using far-UV. Commercially sourced mGH (blue) and the refolded mGH (red) show comparable secondary structure compositions

Far-UV CD spectroscopy was performed to compare the secondary structures of commercially sourced mGH and refolded mGH. mGH is comprised of four antiparallel alpha helices stabilised by disulphide bonds (UniProt ID: P06880). The two proteins assessed both had characteristic minima at 208 nm and 222 nm, which indicates the helicity of the proteins (Fig. 6). The secondary structure of purified mGH (obtained from inclusion bodies with three-cycles of freeze–thaw solubilisation and refolding), closely matched that of the commercial mGH. This alignment confirms that the secondary structure of refolded mGH was preserved. We note that the Far-UV CD spectroscopy appears somewhat noisy, likely due to the relatively low protein concentration used in the measurements.

### In vitro bioactivity of recombinant mGH

**Fig. 7:**
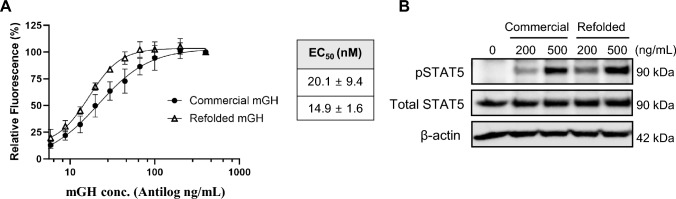
In vitro bioactivity of refolded and purified recombinant mGH. **a** Concentration–response assay comparing refolded GH with a commercial mGH. Ba/F3-mGhr cells were serum-starved and treated with serially diluted concentrations of recombinant mGH. Cells were incubated with mGH for 48 h before determining cell viability with resazurin. The EC_50_ is represented as mean ± SEM of three independent experiments. **b** Western blot analysis of Stat5 phosphorylation (pStat5) as a marker for GHR activation. B16-F10 cells were treated with 200 ng/mL or 500 ng/mL mGH for 30 min and cell lysates were collected

Bioactivity of purified mGH (from the soluble fraction and inclusion bodies) was compared to commercially sourced mGH using a Ba/F3-mGhr dose–response assay. The Ba/F3-mGhr cell line is stably transfected to overexpress mGH receptor and proliferates in response to mGH. Serum-starved Ba/F3-mGhr cells were treated with serial dilutions of mGH (0 – 400 ng/mL) for 48 h. Cell growth was observed in a GH concentration-dependent manner and the EC_50_ values of the refolded and commercial mGH were 14.9 ± 1.6 ng/mL and 20.1 ± 9.4 ng/mL, respectively (Fig. 7A). We also tested the bioactivity of mGH expressed and purified from the soluble fraction in Figure S2 above, and found it had comparable activity to commercial mGH (Figure S4A). However, due to the lower yield only one repeat was carried out and an average EC_50_ was not determined.

Binding of GH to GHR activates the intracellular JAK/STAT5 signalling pathway, whereby STAT5 becomes phosphorylated [11]. Activation of this downstream pathway by refolded mGH was assessed in a mouse melanoma cell line, B16-F10, which expressed the GHR [12]. B16-F10 cells were serum-starved and treated with 200 ng/mL or 500 ng/mL of mGH and incubated for 30 min at 37 °C. mGH (soluble, refolded, and commercial mGH) induced Stat5 phosphorylation with a stronger signal observed with 500 ng/mL mGH (Fig. 7B and Figure S4B).

## Discussion

Methods for expressing and purifying GH from different species have been widely reported [4]. However, there is a paucity of literature detailing techniques for production of mGH and reported methods require specialised equipment. We determined optimal parameters for obtaining a relatively high yield of bioactive recombinant mGH and report a cost-effective method to produce the protein in *E. coli*. In addition, we found that the number of freeze–thaw cycles impacts the solubilisation of Trx-mGH inclusion bodies in 2 M urea. To our knowledge this is the first report of a protein which showed an improvement in yield from inclusion body solubilisation by multiple rounds of freeze–thaw.

Two earlier publications have reported methods for mGH production using the fed-batch fermentation method [5, 6]. Zhou et al*.* described a method for mGH expression and production in GH-NS0 murine myeloma cells using fed-batch culturing for 15–16 days. The final yield was approximately 0.58 g per litre of culture. In the method described by Fradkin et al*.*, recombinant mGH was obtained from *E. coli* grown in fed-batch culture, and protein expressed as inclusion bodies was solubilised and refolded using high pressure solubilisation in 100 mM Tris–HCl, 2 M urea, 1 mM reduced glutathione, 0.1 mM oxidised glutathione, at pH 9. After purification by anion exchange chromatography, the final yield was 3.8 g per litre of culture. In the present study, we explored multiple approaches to effectively obtain active recombinant mGH from *E. coli* using shake flasks, to avoid the need for a specialised fed-batch fermentation system and high-pressure equipment. To enhance solubility and facilitate disulfide bond formation in recombinant mGH, we fused a Trx tag to mGH and expressed it in the AD494 strain.We found that untagged mGH exhibited very low expression levels, whereas Trx-tagged mGH was expressed at much higher levels, suggesting that the Trx fusion enhances expression efficiency in AD494 cells. This improvement may be due to the oxidising cytoplasmic environment of the AD494 strain, which carries a thioredoxin reductase (TrxB) gene mutation that facilitates disulfide bond formation during expression. Overall, the final yield of recombinant mGH expressed from AD494 stain and refolded from inclusion bodies in 2 M urea with three rounds of freeze–thaw was 3.7 mg from 1L culture medium. This yield was tenfold higher than that of mGH purified from the soluble fraction after induction at 18 °C and comparable to the results reported in Fradkin et al*.* utilising a fed-batch culture approach [5].

Endogenous GH is a non-glycosylated protein and is therefore suitable for production in prokaryotic expression systems [13, 14]. However, it accumulates as insoluble protein aggregates in inclusion bodies when expressed in the cytoplasm of *E. coli.* Different approaches to improve soluble expression were attempted in this study. Tagging the protein of interest to Trx is a widely used method to reduce inclusion body formation, and a previous study from our lab reported an improvement in soluble expression in *E. coli* of a mutated version of human GH (B2036) at 18 °C when fused to Trx [15, 16]. Similarly, fusion to Trx resulted in soluble mGH expression when induced at 18 °C but the final yield of mGH was very low (0.3 mg per litre of bacteria culture). In attempt to improve the soluble protein yield, an autoinduction method was employed, as it has been shown to successfully increase the yield of many proteins with minimal intervention [17, 18]. However, this approach did not improve the proportion of soluble Trx-mGH at any point across the total 144 h of cultivation in the autoinduction medium so purification from inclusion bodies was investigated next.

Although the formation of inclusion bodies poses a major hurdle in the recovery of bioactive proteins, it holds certain advantages, such as protection from proteolytic degradation and ease of purification due to the high density of protein [19]. Developing an efficient method to extract biologically active proteins from inclusion bodies is often inevitable for certain proteins. For effective inclusion body solubilisation and recovery, optimisation of multiple parameters is required, such as the denaturant concentration, pH values, and the refolding method [20, 21]. Efficient solubilisation of Trx-mGH was observed with 4, 6, and 8 M urea, at pH 8.5 (Fig. 2A). However, ‘mild’ solubilisation conditions, such as lower denaturant concentrations and alkaline pH, ensure better recovery of the proteins in its conserved native structure, but also helps to avoid protein aggregations during the refolding process [21–24]. We therefore attempted to improve solubilisation using a lower urea concentration (2 M) and a pH value higher than 8.5. Increasing the pH of the 2 M urea buffer from 8.5 to 10.5 substantially increased the proportion of solubilised Trx-mGH protein (Fig. 2B), and no precipitation was observed during the refolding process.

Using a freeze-thawing technique applies physical and chemical stress to the inclusion bodies through the formation of ice crystals, changes in buffer pH, and low temperature. These conditions disrupt hydrophobic interactions and change the role of ionic solvents during protein refolding, thereby denaturing the insoluble proteins [23, 25–27]. Many recent studies requiring inclusion body solubilisation have employed the single round of freeze–thaw strategy to aid solubilisation [7, 28, 29]. The mechanism by which the freeze–thaw method improves protein solubility has been studied by several groups. Under milder conditions (e.g. 2 M urea), proteins are less likely to denature, and these conditions primarily disrupt hydrophobic interactions, which help separate the molecules [30, 31]. In addition, an alkaline pH which is not too close to the isoelectric point (pI) of a protein also plays a crucial role in avoiding protein aggregation. For mGH, with a theoretical pI of 6.37, such conditions (pH 9.5) may facilitate efficient refolding without leading to aggregation. Freezing induces additional stresses that can denature proteins, such as cold temperature, ice crystal formation, salt concentration due to the insolubility of some salts and pH shifts [25]. However, the primary factor contributing to improved solubilisation via the freeze–thaw method is the stress caused by the cold temperature and ice crystal formation during freezing. These factors create localised stresses that disrupt non-covalent interactions, such as hydrophobic and ionic bonds, which hold proteins in aggregated states. Upon thawing, proteins are allowed to refold into their native conformations in a more relaxed environment.

We modified the conventional single-round freeze–thaw method in an attempt to improve the soluble yield. Inclusion body pellets which underwent three rounds of freeze–thaw produced higher amounts of solubilised proteins than those that underwent one round. Furthermore, three rounds of freeze–thaw in 2 M urea had achieved a similar level of solubilisation to that of 8 M urea which typically results in complete solubilisation. This implies that a single round of freeze thaw may be insufficient for some inclusion bodies. We also tried an additional approach for solubilising Trx-mGH where the volume of resuspension buffer (6 mL) was evenly divided over the three freeze–thaw cycles (2 mL per cycle). This approach appeared to slightly improve solubility, although there was variability across experiments due to the nature of the technique so further investigation would be required to confirm this. We also note that this approach was less practical as it involved more handling steps so using three cycles of freeze–thaw without buffer replacement would be a preferable approach.

To our knowledge, this study is the first to report an improvement in inclusion body solubilisation for a protein with repeated freeze-thawing. In a previous investigation which performed a similar experiment to purify EGFP from inclusion bodies, freezing for one day followed by thawing solubilised more inclusion bodies than the traditional denaturing method (no freeze–thaw); but, additional freeze–thaw cycles (up to 4 rounds), or longer freezing durations in the freezer did not improve solubilisation any further [32]. Although there is currently limited research on inclusion body solubilisation for GH, there has been a report discussing the disadvantages of this technique for human GH. This study observed protein aggregation during refolding upon inclusion body freeze–thaw in 2 M urea + 1 mM DTT in 50 mM Tris buffer, which resulted in poor overall recovery and low bioactivity [33]. Although the refolding efficiency was not determined for our mGH production process, repeated freeze-thawing assisted in improving the yield with no observable protein aggregation during refolding, plus it exhibited comparable bioactivities to that of commercial mGH.

The precise mechanism by which repeated freeze–thaw cycles enhance solubilisation is unclear. We hypothesise that the cumulative stress from each cycle progressively disrupts protein aggregates, gradually breaking apart larger inclusion body structures that might resist solubilisation with a single cycle. For some proteins, this stepwise disaggregation likely improves access to the refolding environment, reducing aggregation risks and promoting the recovery of functional protein. The solubilisation conditions and the multiple-freeze–thaw approach are highly protein-dependent, and we recommend researchers desiring to produce recombinant proteins from inclusion bodies to explore such methods for optimised protein recovery, if not already published in literature.

The methodology described here provides a quick, low-cost, and convenient alternative approach for purifying mGH for laboratory use. However, optimisation of additional parameters might improve the yield further. These may include hydrostatic pressure [34], the inclusion of organic solvents such as trifluoroethanol or *n*-propanol [22, 35], and the use of extreme pH conditions [23, 36–38], all of which have shown potential in improving solubilisation and refolding efficiency in related systems [39].

## Conclusions

This study describes an efficient and cost-effective method for producing mGH protein using *E. coli* without the need for specialised fermentation and pressure-refolding equipment. Solubilisation of mGH protein improved considerably when mGH inclusion bodies underwent multiple freeze–thaw cycles compared to a single cycle, and resulted in more than a tenfold improvement in final yield compared to soluble expression at 18 °C. This approach may also have application for other proteins with low yield following a single round of inclusion body freeze–thaw.

## Supplementary Information

Below is the link to the electronic supplementary material.

Supplementary file1 (PDF 1168 KB)

## Data Availability

All data generated and analysed during this study are included in this manuscript and its supplementary materials.
