# Supplementary material for: Recovery of mouse growth hormone from E. coli inclusion bodies using a mild solubilisation and repeated freeze–thaw approach
Source: Mol Biol Rep. 2025 Jun 23;52(1):627. doi: 10.1007/s11033-025-10685-y (PMC12185587; doi:10.1007/s11033-025-10685-y)
Supplement: Supplementary file 1 — Supplementary file1 (PDF 1168 KB) [file 11033_2025_10685_MOESM1_ESM.pdf]

Applied Microbiology and Biotechnology

Recovery of mouse growth hormone from *E. coli* inclusion bodies using a mild solubilisation approach

Minah Kim[1], Ries J. Langley[2,3], Jo K. Perry[1,3*], Yue Wang[1,3*]

Authors' affiliations:

[1] Liggins Institute, University of Auckland, Auckland, New Zealand, 1023

[2] Department of Molecular Medicine and Pathology, University of Auckland, Auckland, New Zealand, 1023

[3] Maurice Wilkins Centre for Molecular Biodiscovery, Auckland, New Zealand, 1023

*Co-corresponding authors:

Dr Yue Wang, PhD

Email: wang.yue@auckland.ac.nz

ORCID: 0000-0001-9010-4177

and

Associate Professor Jo. K. Perry, PhD

Email: j.perry@auckland.ac.nz

The Liggins Institute, University of Auckland

85 Park Rd, Private Bag 92019 Auckland 1142, New Zealand

Tel: +64(9) 9237873; Fax: +64(9) 3737497

Insert sequence

TTCCCCGCTATGCCGCTGTCATCGCTCTTCTCAAATGCAGTTCTC
CGAGCGCAACATCTTCATCAATTGGCGGCCGACACCTACAAAGAG
TTCGAGCGGGCCTATATCCCAGAAGGCCAACGCTATAGCATACAA
AACGCCCAGGCTGCTTTCTGTTTTAGCGAGACTATTCCTGCGCCA
ACTGGAAAAGAAGAGGCTCAGCAGCGCACGGATATGGAGTTGTTA
CGCTTTTCCTTGTTGCTTATCCAGTCATGGTTAGGACCGGTTCAG
TTCTTGTCAAGAATTTTCACAAATAGCTTAATGTTCGGCACGTCC
GACCGCGTATACGAGAAATTAAAAGACCTTGAGGAAGGGATACAA
GCTCTGATGCAAGAGTTAGAGGATGGCTCGCCACGAGTAGGTCAA
ATACTTAAACAAACTTATGATAAGTTTGATGCGAATATGCGTTCT
GACGACGCACTTCTGAAGAATTATGGTCTGCTCTCTTGCTTCAAG
AAAGATCTGCACAAGGCCGAGACATATTTGCGAGTGATGAAGTGT
AGAAGATTCGTCGAGTCCTCATGCGCTTTTTAATGA

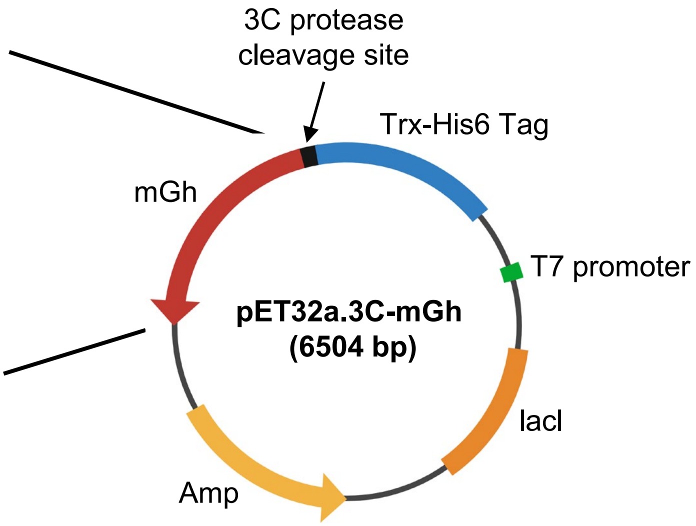

mGH amino acid sequence

GPGSFPAMPLSSLFSNAVLRAQHLHQLAADTYKEFERAYIPEGQR
YSIQNAQAAFCFSETIPAPTGKEEAQQRTDMELLRFSLLLIQSWL
GPVQFLSRIFTNSLMFGTSDRVYEKLKDLEEGIQALMQELEDGSP
RVGQILKQTYDKFDANMRSDDALLKNYGLLSCFKKDLHKAETYLR
VMKCRRFVESSCAF

Primer sequences

- Forward primer: 5'-CGCGGATCCTTCCCCGCTATGCCGCTGTC-3' (underline denotes *Bam*HI)
- Reverse primer: 5'-CGCGAATTCTCATTAAAAAGCGCATGAGG-3' (underline denotes *Eco*RI)

Figure S1. Schematic diagram of the pET32a.3C-mGh plasmid construct. The *mGh* gene sequence was optimised for expression in *E. coli* and synthesised by TWIST Bioscience. The insert was amplified by PCR using the forward and reverse primers (forward: 5'-CGCGGATCCTTCCCCGCTATGCCGCTGTC-3'; reverse: 5'-CGCGAATTCTCATTAAAAAGCGCATGAGG-3') containing *Bam*HI and *Eco*RI restriction enzyme sites (underlined). Fragments were digested with *Bam*HI and *Eco*RI and cloned into a modified pET32a.3C vector which contains a human rhinovirus 3C protease cleavage site after the N-terminal Thioredoxin-His-6 tag. Four additional amino acids remain at the N-terminal of the mGH protein after 3C cleavage (underlined).

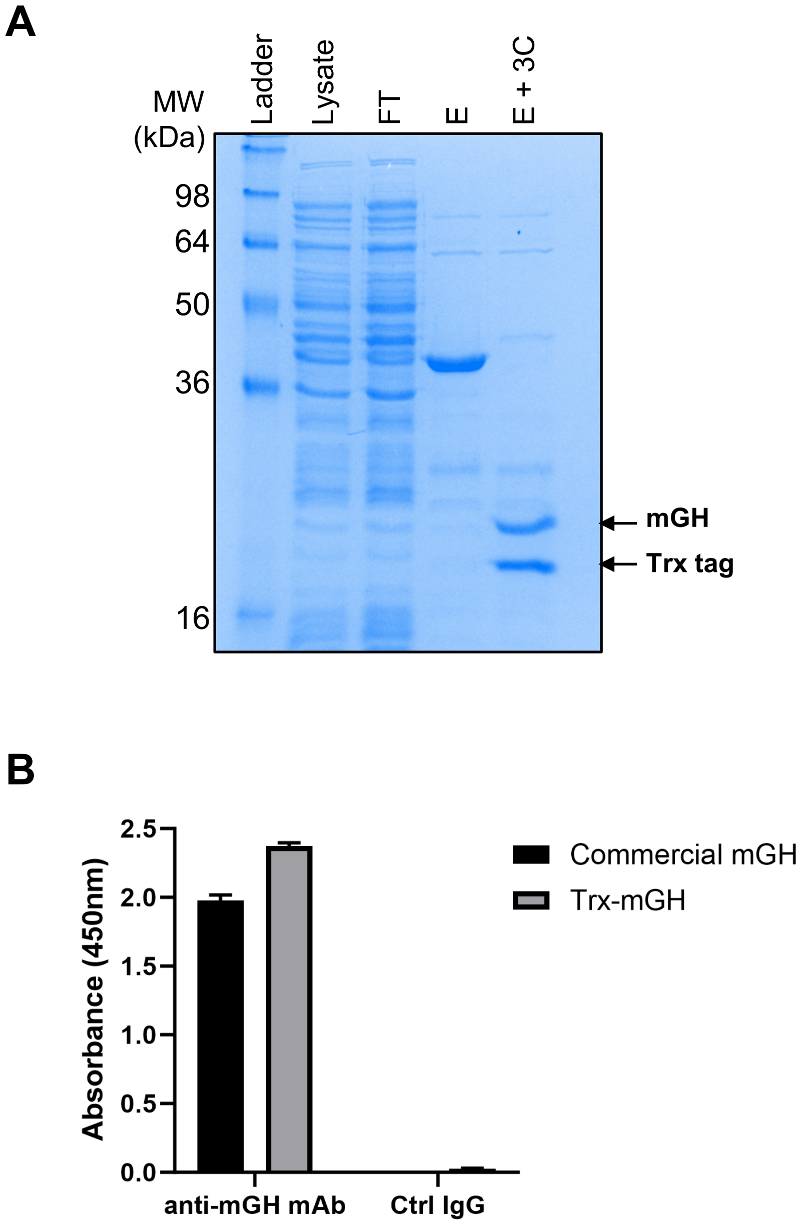

Figure S2. Purification of soluble recombinant mGH. *E. coli* harbouring the pET32a.3C-mGh plasmid were induced with IPTG at 18°C. The supernatant of the bacterial lysates was collected and purified using Ni-NTA. (A) SDS-PAGE analysis of the total protein in bacterial lysates (Lysate), the flow-through fraction containing all proteins without the Trx-tag (FT), the elution fraction containing Trx-mGH (E), and Trx-mGH cleaved by 3C protease (E+3C). (B) ELISA using an in-house anti-mGH monoclonal antibody (mAb) and control IgG (Ctrl IgG). Microtiter plates were coated with mGH and Trx-mGH at 2 µg/mL and 4 µg/mL, respectively, and primary antibody concentration was 2 µg/mL. Ctrl IgG was used as negative control. Values are mean ± SD of n = 2 replicates.

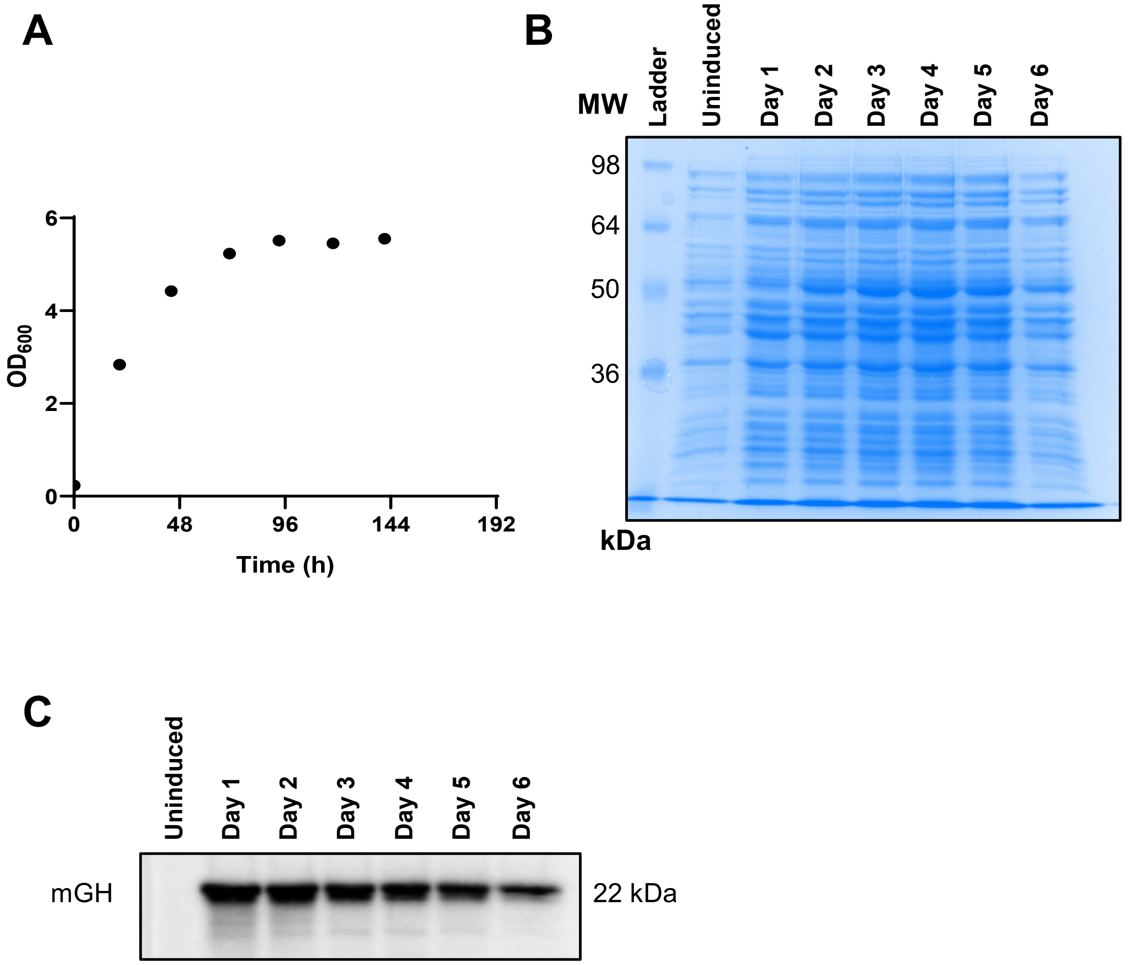

Figure S3. Analysis of soluble mGH expression in *E. coli* grown in autoinduction media. (A) *E. coli* harbouring pET32a.3C-mGh plasmid were grown in AIM-2YT Broth (Formedium Ltd.) and OD_{600} measurements were taken periodically over 144 h. (B) SDS-PAGE analysis of soluble mGH protein over 6 days. (C) Western blot detecting mGH in bacterial lysates collected from the autoinduction culture on each day. Equal amounts of total protein were loaded in each lane.

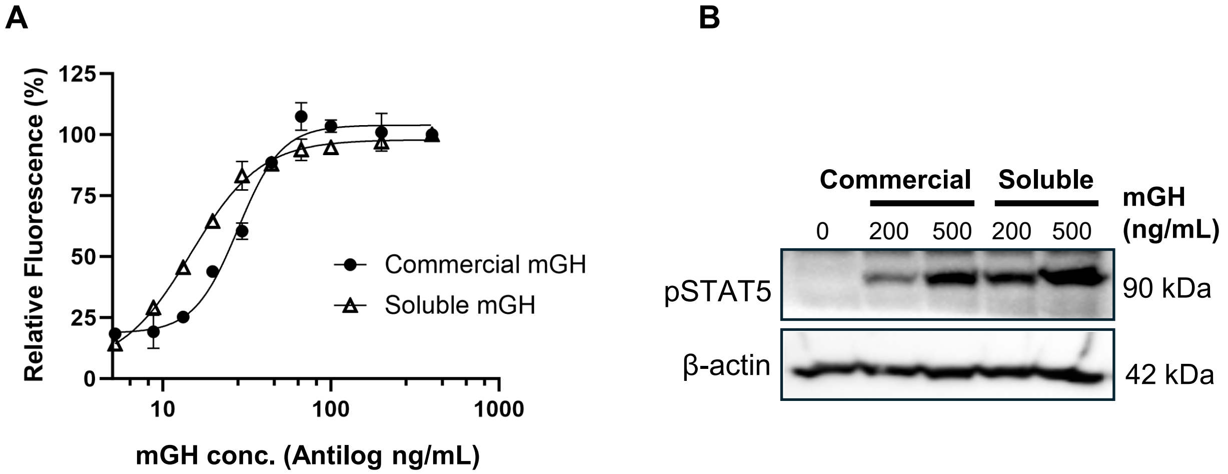

Figure S4. *In vitro* bioactivity of soluble recombinant mGH. (A) Concentration-response of soluble mGH compared to a commercial mGH. Ba/F3-mGhr cells were serum-starved and treated with serially diluted concentrations of recombinant mGH. Cells were incubated with mGH for 48 h before determining cell viability with resazurin. (B) Western blot analysis of STAT5 phosphorylation (pSTAT5) as a marker for GHR activation. B16-F10 cells were treated with 200 ng/mL or 500 ng/mL commercial or soluble mGH for 30 min and cell lysates were collected. Due to the low yield of soluble mGH only one experimental repeat was conducted and an average EC_{50} was not determined.
